# Implementation and utilization of gynecological teaching associate and male urogenital teaching associate programs: a scoping review

**DOI:** 10.1186/s41077-021-00172-2

**Published:** 2021-05-20

**Authors:** Holly Hopkins, Chelsea Weaks, Elise Napier

**Affiliations:** 1 Eastern Michigan University School of Nursing, 311 Marshall Building, Ypsilanti, MI 48197 USA; 2 Standardized Patient Educator, GTA Program, Eastern Virginia Medical School Sentara Center for Simulation and Immersive Learning, 651 Colley Avenue, PO Box 1980, Norfolk, VA 23501-1980 USA; 3 Ferris State University, 1201 S. State Street, Big Rapids, MI 49307 USA

**Keywords:** Gynecological teaching associate, Male urogenital teaching associate, Genitourinary teaching associate, Standardized patient, Professional patient, Standardized patient methodology, Physical examination instruction, Pelvic examination, Genitourinary examination, Rectal/prostate examination

## Abstract

**Background:**

Gynecological Teaching Associates (GTAs) and Male Urogenital Teaching Associates (MUTAs) are individuals trained to instruct health professional learners with their own body to conduct accurate, patient-centered breast, pelvic, urogenital, rectal, and/or prostate examinations. Evidence indicates that this results in improvements in technical competence and communication skills, but there is wide variability to how such programs are implemented and engaged within the curriculum. In this scoping review, we mapped evidence regarding (1) how GTA/MUTA programs are utilized with health professional learners, (2) how GTA/MUTA programs are implemented using the Association of Standardized Patient Educators (ASPE) Standards of Best Practice (SOBP) as a framework, and (3) what broad outcomes are addressed in publications.

**Methods:**

PubMed, ERIC, PsychINFO, CINAHL, and Sociological Abstracts were searched for all publications addressing instruction of physical examinations with a GTA/MUTA and/or administration of GTA/MUTA programs. Studies were charted in tandem until consensus was identified and then charted individually, using an iterative process. The scoping review protocol was registered prospectively.

**Results:**

One hundred and one articles were identified, and nearly all highlighted positive results regarding GTA/MUTA programs. Most studies addressed medical students within the USA and Europe. During instructional sessions, three (SD=1.4) learners worked with each GTA/MUTA and an average of 32 min (SD=17) was allocated per learner. GTAs/MUTA instructed both independently (n=33) and in pairs (n=51). Thirty-eight articles provided detailed information consistent with one or more of the Domains of the ASPE SOBP, with six providing specific information regarding safe work environments.

**Conclusions:**

While studies demonstrate consistently positive outcomes for learners, there is wide variability in implementation patterns. This variability may impact learning outcomes and impact both physical and psychological safety for GTAs/MUTAs and learners. Terminology used to refer to GTAs/MUTAs is inconsistent and may obscure relevant publications. Additional research is indicated to explore the pedagogical variables that result in positive learning outcomes and examine methods to ensure physical and psychological safety of GTAs/MUTAs and learners.

**Trial registration:**

https://osf.io/x9w2u/.

**Supplementary Information:**

The online version contains supplementary material available at 10.1186/s41077-021-00172-2.

## Background

Gynecological Teaching Associates (GTAs) “teach, assess, and provide feedback to learners about accurate pelvic, rectal and/or breast examination techniques. They also address the communication skills needed to provide a comfortable exam in a standardized manner, while using their bodies [to instruct] in a supportive, non-threatening environment” [1]. Male Urogenital Teaching Associates (MUTAs) use the same methodology to educate learners on the urogenital and rectal examination [1]. GTAs instruct breast examinations in 65% and pelvic examinations in 72% of US medical schools [2]; consistent with a report in 1983 illustrating that 78% of US medical schools engaged teaching associates to instruct pelvic examinations [3]. Both studies found that the pedagogical implementation of these methodologies varied widely [2, 3], which was also indicated by van Ravensteijn, Hageraats, and Rethans [4], but has not been examined on a broad scale. Similar data for MUTAs is much more limited [5].

The seminal work on GTA methodology grew from the founder’s experience with standardized patient (SP) methodology [6], and GTAs/MUTAs continue to represent an application of SP methodology [7, 8]. SPs may teach and assess physical examination skills as a part of their role, which may also include role portrayal [1]. GTAs, MUTAs, and SPs share the same underlying values [7, 9]; however, the GTA/MUTA role is uniquely focused. While receiving an examination, the GTA/MUTA is in the position of the “patient” while continuing to engage in real-time instruction throughout that experience; in most circumstances, this reduces the opportunity for role portrayal as may be expected of an SP.

Two systematic reviews have been conducted in relation to GTAs. Jha, Setna, Quinton, and Roberts [10] evaluated evidence on the engagement of SPs and/or real patients in the instruction of breast, pelvic, urogenital, and/or breast examination techniques finding that most of the studies demonstrated short-term beneficial outcomes regarding technical competence and satisfaction. Little evidence was found regarding program sustainability, implementation, or long-term outcomes for learners. Smith, Choudhury, and Clark [11] identified that learner competence and communication skills were improved following a GTA session, but there was no impact on confidence. No similar reviews could be identified regarding MUTAs. No additional systematic reviews or scoping reviews have been identified related to GTA and MUTA work. Nevertheless, within the last 11 years, there have been additional publications addressing GTA/MUTA programs with the ASPE Standards of Best Practice (SOBP) [7] and the ASPE GTA/MUTA SOBP [9] providing a growing framework for program implementation and GTA/MUTA engagement. Developing a broader understanding of GTA/MUTA program utilization and implementation will help to advance the field, continue to shape relevant SOBP, and identify areas of research to uncover aspects of GTA/MUTA programs that best facilitate learning.

The objective of this scoping review is to map the available evidence regarding implementation and utilization of GTA and MUTA programs in the education of health professional students, in alignment with Preferred Reporting Items for Systematic Review and Meta-Analyses extension for Scoping Review (PRISMA-ScR) guidelines [12]. The following research questions will be addressed:

- How are GTA and MUTA programs utilized within the education of health professional learners?
- How are GTA and MUTA programs implemented?
- What broad outcomes do GTA and MUTA publications address?

## Methods

The protocol was drafted using the Preferred Reporting Items for Systematic Reviews and Meta-Analysis Protocols (PRISMA-P [13];) and registered prospectively with the Open Science Framework [14].

Articles were included if they discussed (1) instruction of breast, pelvic, rectal, and/or urogenital examination with a GTA/MUTA or (2) administration of a GTA/MUTA program. No limitations were placed on study methodology or timeframe, but the full text had to be available in the English language. Articles where a healthcare professional was providing all instruction on a live human model who provided no feedback were excluded, as were Letters to the Editor that did not substantially contribute to the field.

**Fig. 1:**
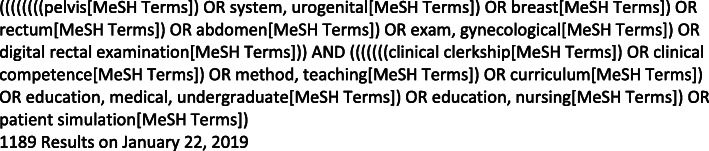
PubMed search strategy

PubMed, ERIC, PsychINFO, CINAHL, and Sociological Abstracts were searched on January 23, 2019. PubMed and CINAHL, as the search engines with the most relevant articles, were searched again on May 22, 2020, to identify any recent publications. The search strategy used in PubMed is available in Fig. 1. Search results were uploaded into Covidence [15] for screening. Reference lists for the included articles were hand-searched for relevant citations.

### Selection of sources of evidence

#### Step 1

Each title and/or abstract was reviewed by an independent reviewer to determine whether inclusion criteria were met. The first thirty titles and/or abstracts were reviewed by two randomized reviewers to evaluate consistency among reviewers. Uncertainties and disagreements were resolved through discussion and group consensus. Once consensus was identified, the remaining articles were divided among the three reviewers who each screened independently.

#### Step 2

All articles that met inclusion criteria for step 1 were reviewed in full. The first twenty full-text articles were reviewed by two randomized reviewers to evaluate consistency among reviewers. Uncertainties and disagreements were resolved through discussion and group consensus. Once consensus was identified, the remaining articles were divided among the three reviewers who each screened independently.

#### Step 3

Reference lists of included articles were hand-searched for additional relevant articles.

### Data charting process

Data charting was completed within Google Forms/Sheets [16]. The first nineteen articles were charted by two randomized reviewers and results compared. Disagreements, primarily minor, were resolved through discussion and group consensus. Once consensus was achieved, articles were divided among the three reviewers who each charted the results of included articles through an iterative process whereby previously charted articles were reviewed for modifications to charting when modifications to the template were made. Critical appraisal of individual sources was beyond the scope of this scoping review. Study authors were not contacted for additional details regarding their work.

We charted data on each study’s characteristics (e.g., country of origin, GTA or MUTA focus, terminology, number of GTAs/MUTAs), structure (e.g., session components, type of instruction [independent vs paired], learner type, structure of session, number of learners, session length, timing within curriculum, length of GTA/MUTA training), implementation (degree to which the Domains of the ASPE SOBP [8] were addressed), and broad outcomes (e.g., study aim, learner outcomes, GTA/MUTA outcomes). Tables summarizing all 101 articles are included in the Online Supplementary Materials, with results organized by research question.

## Results

**Fig. 2:**
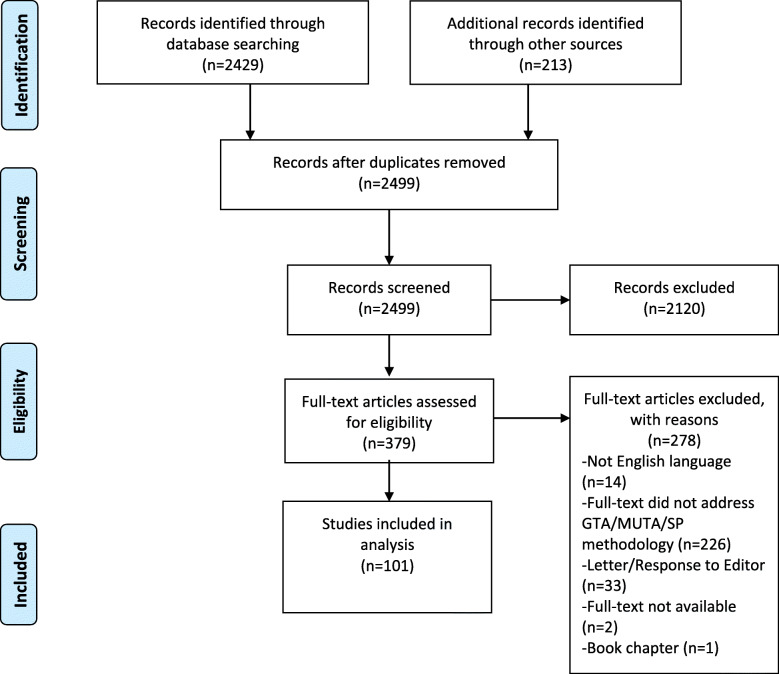
PRISMA flow diagram

**Fig. 3:**
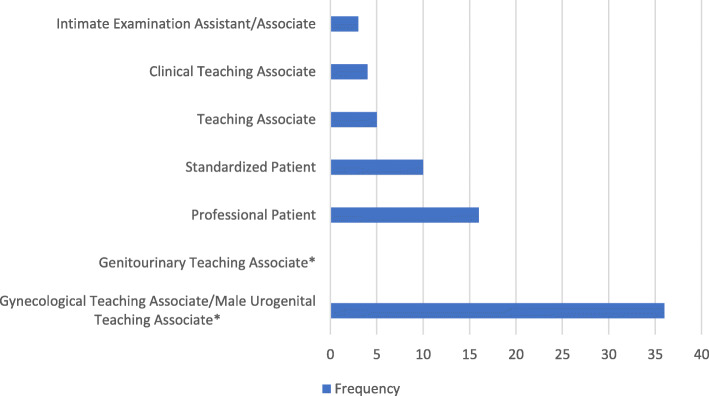
Most frequent terms used to describe GTAs/MUTAs. Asterisk indicates the terms included in the SSIH Dictionary [1]

One hundred and one studies met inclusion criteria (Fig. 2 and Online Supplemental Material: Additional file 1). Twenty-eight studies originated from Europe while 56 took place in the USA. MUTA programs were discussed in 22 studies, 14 of which also addressed GTA programs. Despite a reduction in publication in the 1990s, the number of relevant studies published increased each year. Figure 3 demonstrates the most common terms used to describe GTAs/MUTAs.

Thirty-eight of these studies addressed the number of GTAs/MUTAs involved in the study and/or program, with an average of 10 GTAs/MUTAs per study. Nearly all studies (n=99) reported positive outcomes, although such outcomes were variously defined. While many studies addressed a combination of outcomes, learner outcomes (n=72) were the most common, followed by GTA/MUTA program outcomes (n=34), and GTA/MUTA individual-level outcomes (n=14). The studies on GTA/MUTA program outcomes included reports on unique programs, literature reviews, and surveys of several programs. This breakdown of outcomes is in alignment with the variety of study aims (see Online Supplemental Material: Additional file 2).

### Program utilization and GTA/MUTA engagement

**Fig. 4:**
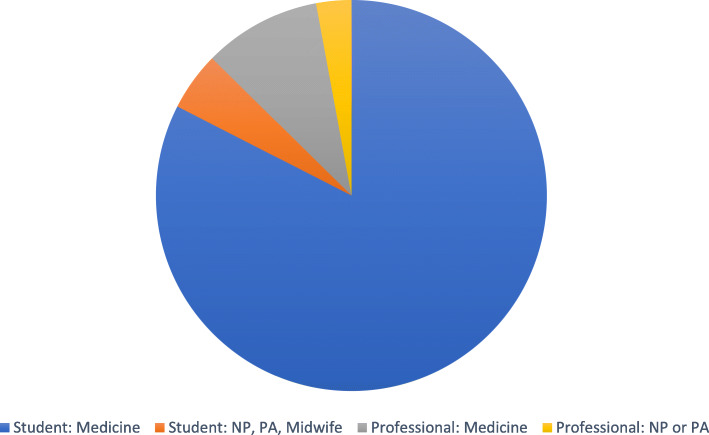
Learners involved in GTA/MUTA instructional sessions

GTAs/MUTAs most frequently work with medical students (see Fig. 4). Although not all studies addressing medical students reported the location within the curriculum (n=20), the second and third years of the curriculum was most common (n=34 and n=27, respectively), with several institutions offering these sessions multiple times during the curriculum (n=17).

Medical students in the second year of their curriculum were most commonly instructed by GTAs/MUTAs. Time per learner (M=32 min, SD=17) was addressed in 48 studies whereas the number of learners per session (M=3.2, SD=1.4, n=70) and session length (M=96 min, SD=53, n=54) were more frequently quantified. Time allocated per learner varied by the exam being taught (breast exam=25 min, n=6; pelvic exam=31 min, n=20; breast and pelvic exam=43 min, n=8; breast, pelvic, and rectal exam=33.75 min, n=2; pelvic and rectal exam=32.5 min, n=2; rectal exam=24 min, n=3; urogenital and prostate/rectal exam=32.5 min, n=3). GTAs most commonly taught pelvic examination techniques (n=75) followed by breast examination (n=35) and female rectal examination (n=13). MUTAs most commonly taught the male rectal/prostate examination techniques (n=21) and urogenital examination (n=17). Many studies incorporated additional details such as patient-centered communication techniques and eliciting a patient’s history, but details were insufficient to measure and quantify across studies.

Thirty-three studies presented GTA/MUTA programs in which the GTA/MUTA instructed independently; 51 studies presented methods of paired instruction with either a peer GTA/MUTA or healthcare professional (most commonly a physician). The details of the paired instruction methods were variable: some programs had the GTA/MUTA experiencing the exam portray the “patient” role while the other GTA/MUTA portrays the “provider” role, demonstrating an exam and guiding learners; other programs had a second person (e.g., GTA/MUTA or faculty) in the room to function only as a resource or chaperone.

Of the 18 studies addressing training, the mean training duration for novice GTAs/MUTAs was 15 h (SD=10). Studies presenting their training in a matter of days and/or weeks were removed from this analysis due to ambiguity. Pay was addressed infrequently. Ten studies reported pay by the hour, but ranges were generally non-specific and/or widely variable across time and currency; these limitations resulted in omission from Online Supplemental Material: Additional file 3.

### Implementation

**Fig. 5:**
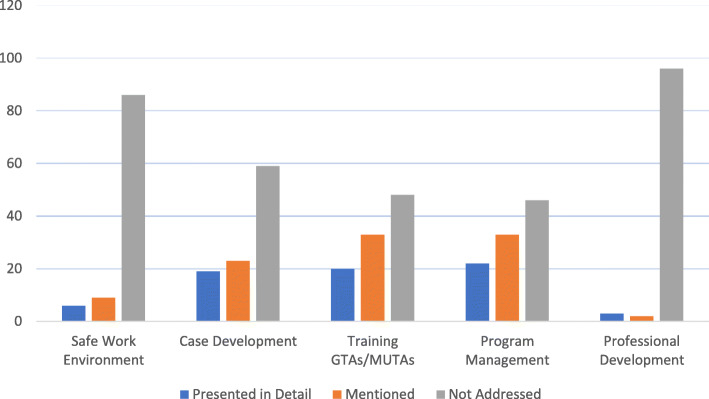
Implementation of GTA/MUTA programs. Studies were compared to the five Domains of the ASPE SOBP [7]. “Presented in Detail” indicates the number of studies that presented sufficient information to replicate components of that Domain. “Mentioned” indicates the number of studies that reported engaging in a Domain, but did not provide sufficient information for replication (e.g., “the GTAs were trained” without further details). “Not Addressed” indicates the number of studies that did not address the Domain

When compared to the five Domains within the ASPE SOBP [7], thirty-eight articles provided a detailed programmatic analysis related to one or more Domain(s) (Fig. 5 and see Online Supplemental Material: Additional file 4).

## Discussion

Despite publications presenting MUTA programs [17, 18] being released within years of the report of the first GTA program [19, 20], most articles continue to focus primarily on GTAs. Even the Healthcare Simulation Dictionary [1] incorporates MUTAs as a subset of GTAs, rather than a distinct or analogous role. Is there a reason for this discrepancy? Although the anatomy and relevant examination techniques vary, program descriptions and definitions indicate that the structure and intention of MUTA sessions are otherwise very similar to GTA sessions. This suggests that many implementation and utilization characteristics can likely be applied to both GTA and MUTA programs. Further research is recommended to collect additional evidence regarding MUTA programs and to identify similarities and differences between GTA and MUTA programs.

Program descriptions also indicate that engagement patterns are similar in spite of wide variation in terminology. These variations in terminology challenge research efforts and obscure potentially beneficial publications. Kretzschmar’s [6] landmark publication coined the terms “Gynecology Teaching Associate” and “Professional Patient,” and the official definitions for GTA, MUTA, and GUTA have existed for years. Upholding standardized terminology ensures a shared understanding of the concepts being discussed [21]. It is not uncommon for programs that engage patient models for demonstration to identify themselves as a “GTA/MUTA” program despite the individual receiving the physical assessment being a passive receiver of an examination, as opposed to instructing the examination [22]. This hinders effective communication, reduces the impact of relevant research, and poses a substantial risk for harm if inappropriate methodologies are unknowingly applied due to misunderstandings. Consistent use of terminology in alignment with the Healthcare Simulation Dictionary [1] and application of the ASPE GTA/MUTA SOBP [9] will help support clarity on this methodology while facilitating a safe, effective teaching/learning environment.

### Program utilization and engagement

Although much work has been done to assess the learner’s response to the program (which is consistently positive), the characteristics that make GTA/MUTA programs most valuable have yet to be sufficiently identified and articulated. For example, GTA/MUTA preparation, learner objectives, learner preparation, and time allocated to the session will impact learner outcomes, but the importance of these individual variables is not yet known or fully understood. In most studies, these variables were either not explicit or insufficiently specific to evaluate. Many studies report that the GTAs/MUTAs provide “feedback” or “instruction,” yet both of those processes can be undertaken in a variety of methods. Real-time feedback during a session (e.g., confirmation of comfort, technique, and anatomy being palpated) is distinct from completion of a checklist on conclusion of the session. Adhering to the publication recommendations to enhance the quality of Standardized Patient research [23] will enhance the rigor of GTA/MUTA publications.

There is some evidence exploring use of task trainers before and after GTA sessions [24], but there is otherwise very little exploration of GTA/MUTA pedagogy. An instructional session with the objective of developing learner competence in visual identification of the cervix may (or may not) need more time than a session with the objective of placing a speculum. A MUTA program whose central objective is to identify a healthy prostate may (or may not) choose to employ MUTAs with ongoing prostate pathology. A GTA/MUTA who instructs independently may (or may not) support the learner’s belief that the “patient” is in control of their clinical encounter as opposed to the faculty or the examiner. Learners are being allocated between 10 and 75 min for instruction on the pelvic examination, which is most widely reported. These programs are almost exclusively used in graduate-level health professions programs, where effective pedagogy is essential for optimizing learning outcomes and later patient outcomes. While experiential learning [25] is being prioritized, the wide variance described in program utilization illustrates that we have not yet identified ways to optimize learning outcomes using GTA/MUTA methodology. While this may be challenging to explore, enhanced reporting consistent with Howley et al. [23] will facilitate sharing of such data.

### Implementation

The ASPE SOBP [7] was the first document to illustrate standards of best practice for Standardized Patient Educators. Some of the Principles and Practices do not translate perfectly to GTA/MUTA programs, so the spirit of the document was used in this study to guide assessment of implementation characteristics. GTAs and MUTAs represent an application of Standardized Patient methodology so while the original ASPE SOBP has been applied in many contexts, the ASPE GTA/MUTA SOBP [9] demonstrates the Principles and Practices specific to offering instructional sessions that engage GTAs/MUTAs. These documents should help researchers better identify program characteristics to implement and report upon thus elevating the rigor of future publications and the field as whole.

### Ethical considerations and humanization

This instructional methodology grew out of significant ethical concerns regarding the common practice of incorporating sex workers and/or unconsented anesthetized clients into instructional settings [26–29]. GTAs/MUTAs generally consent to their work; nevertheless, opportunities for coercion and harm remain [30]. For example, testimony in 2019 alleged misconduct including coercion and sexual harassment by administrators against clinical models for breast, pelvic, and rectal examinations [31]; this lawsuit was filed in the wake of the investigations into the even more egregious conduct of Dr. Larry Nassar. While this is an extreme circumstance involving patient models as opposed to GTAs, it suggests that administrative structures and mechanisms that can foster or enable coercive or other unethical behaviors may still be a serious issue in GTA/MUTA programs.

These practices highlight the critical importance of robust administrative processes including recruitment, hiring, and provision of rigorous training to support GTAs/MUTAs becoming effective instructors. Poor practices may increase the risk of harm to GTAs/MUTAs such as having poorly defined expectations; having poorly prepared or unprofessional learners; and placing the GTA/MUTA in a position where another individual in the instructional session holds greater power than they do, or a position where they are/perceived to be unable to decline work (in part or in whole).

Sexual harassment is common within medical schools [32–35] and healthcare facilities both in the USA [36] and abroad [37]. In the USA, the broader population reports 44% of women and 25% of men experiencing some form of sexual violence in their life, often before age 25 [36]. Worldwide, sexual and/or intimate partner violence impacts 35% of women [37]. In some studies within healthcare settings, faculty and healthcare professionals are more commonly reported to be perpetrators, compared to patients and their families [32, 33]. Instructional sessions therefore have high potential to include both survivors and perpetrators of sexual harassment, sexual violence, and other forms of violence. Although this risk, along with steps to address it, may be made explicit in some training programs, it is not well-addressed in the literature. Many opportunities exist to promote safety in these unique instructional settings, such as effective preparation of learners and GTAs/MUTAs with clear guidelines; inclusion of a chaperone (often additional learner(s)); provision of secure learning environments; and connection to relevant resources on campus.

One survey asked GTAs if they “felt ‘used’”; fortunately they did not, but this is a legitimate concern [38] that requires continued vigilance. Research and oversight should be conducted to ensure that GTA/MUTA programs maintain high ethical standards, and guard against the ethical issues similar to those that brought forth this methodology. The ASPE SOBP [7] and ASPE GTA/MUTA SOBP [9] provide effective frameworks to aid in prevention of such issues, particularly within the Domain addressing Safe Work Environment. This is essential for programs to address, yet is not frequently explored within the included studies.

By addressing these concerns, we can emphasize the safe practice of these skills as they relate to the learner’s future patients. While GTAs/MUTAs are not typically portraying a patient role during their instructional session, they are human and so experience the same sensations, and potentially harms, as a patient may when receiving care. Making the transition from seeing the GTA/MUTA as an educational tool and focusing more on their experience as humans who instruct with their body may help facilitate a shift in the ethical or moral perspectives used to guide this work [30, 39]. Safe work practices that maintain GTA/MUTA safety are, in many ways, consistent with patient safety recommendations. Protecting GTAs/MUTAs demonstrates not only respect regarding their human experience of instructing but also demonstrates the importance of patient safety for the learners they instruct. This may include empowering the GTA/MUTA to pause a session at any time for any reason or may be as complex as considering the maximum number of examinations a GTA/MUTA can instruct or receive in any timeframe. The latter represents a critical question that is commonly discussed within GTA/MUTA programs and organizations [22]—there are challenges in balancing efficiency and the human experience that must be navigated. For example, with repeated palpation of the same structure, the sensation of that exam and/or the anatomy itself may change. Additional research is recommended to evaluate the number of exams that a GTA/MUTA may safely experience in a given timeframe and to identify techniques that may be incorporated to enhance both physical and psychological safety for the GTA/MUTA.

Several studies address, at least in part, the learner experience within a GTA/MUTA session, yet few address the characteristics of GTAs/MUTAs and how they are individually impacted by their work (e.g., [40–44]). Additional research is indicated to explore motivations and experiences of GTAs/MUTAs.

### Limitations

It is possible that publications have been omitted due to variations in terminology and/or indexing. GTA/MUTA methodology has been reported in several textbooks [8, 45] and books [27], which were not included in this review due to logistical constraints. Researchers charting data in tandem (two at the same time) may enhance reporting; however, consensus was reached quickly and this is not believed to be a significant challenge. Reporting of various details of GTA/MUTA program utilization and implementation may be incomplete and does not necessarily reflect the breadth of GTA/MUTA programs that exist globally.

## Conclusion

This scoping review is not intended to provide recommendations on GTA/MUTA program structure as there are many contextual variables that inform decisions at the local level. This study is rather intended to map the current state of the literature, which has been accomplished. Studies about GTA/MUTA methodology have demonstrated overwhelmingly positive learner outcomes for over 60 years, yet there is wide variability in how the studies are presented in the literature. Use of standardized terminology [1] in studies that incorporate the ASPE GTA/MUTA SOBP [9], the ASPE SOBP [7] as indicated, and adhere to SP research publication recommendations [23] are critical to advancing this field. Potential research questions have been identified and the authors hope that future work will angle the lens to more closely examine (1) physical and psychological safety and (2) GTA/MUTA program characteristics that result in positive outcomes.

## Supplementary Information

**Additional file 1.** Title of Data: Studies Meeting Inclusion Criteria.

**Additional file 2.** Title of Data: Broad Outcomes of GTA/MUTA Studies.

**Additional file 3.** Title of Data: Utilization of GTA/MUTA Programs.

**Additional file 4.** Title of Data: Implementation of GTA/MUTA Programs.

## Data Availability

Not applicable, all data are available within Supplemental Files.

## Abbreviations

ASPE: Association of Standardized Patient Educators
GTA: Gynecological teaching associate
GUTA: Genitourinary teaching associate
MUTA: Male urogenital teaching associate
SOBP: Standards of best practice
