# Supplementary material for: Implementation and utilization of gynecological teaching associate and male urogenital teaching associate programs: a scoping review
Source: Adv Simul (Lond). 2021 May 20;6:19. doi: 10.1186/s41077-021-00172-2 (PMC8138924; doi:10.1186/s41077-021-00172-2)
Supplement: Supplementary file 1 — Additional file 1. Title of Data: Studies Meeting Inclusion Criteria. [file 41077_2021_172_MOESM1_ESM.pdf]

Online Supplementary Materials
Studies Meeting Inclusion Criteria

1. Abraham A: Vaginal and speculum examination in medical curricula. Aust NZ J Obstet Gynaeol 1995, 35,56-60.
2. Abraham S: Gynaecological examination: a teaching package integrating assessment with learning. Medical Education 1998, 32, 76-81.
3. Abraham S, Chapman M, Taylor A, McBride A, Boyd C: Anxiety and feelings of medical students conducting their first gynecological examination. Journal of Psychosomatic Obstetrics & Gynecology 2003, 24(1),39-44.
4. Barnard A, Owen C, Tyson A, Martin S: Maximising student preparation for clinical teaching placements. The Clinical Teacher 2011, 8,88-92.
5. Barrett SV, Sapka JG, Mazor KM, Luckmann RL: Assessing third-year medical students' breast cancer screening skills. Academic Medicine 2002, 77,905-910.
6. Beckmann CRB, Barzansky BM, Sharf BF, Meyers K: Training gynaecological teaching associates. Medical Education 1988, 22:124-131.
7. Beckmann CRB, Lipscomb GH, Williford :, Bryant E, Ling FW: Gynaecological teaching associates in the 1990s. Medical Education 1992, 26,105-109l.
8. Beckmann CRB, Meyers K: Mental and physical effects of being a Gynecologic Teaching Associate. The Journal of Reproductive Medicine 1988, 33(1),22-24.
9. Beckmann CRB, Sharf BF, Barzansky BM, Spellacy WN: Student response to gynecologic teaching associates. Am J Obstet Gynecol 1986, 155, 301-306.
10. Beckmann CRB, Spellacy WN, Yonke A, Barzansky B, Cunningham RP: Initial instructional in the pelvic examination in the United States and Canada, 1983. American Journal of Obstetrics and Gynecology 1985, 151(1),58-60.
11. Behrens A, Barnes HV, Gerber WL, Albanese M, Matthes S, Cangelosi A: model for teaching sophomore medical students the essentials of the male genital-rectal examination. Journal of Medical Education 1979, 54:585-587.
12. Biggs JSG, Harden RM, Howie P: Undergraduate obstetrics and gynaecology in the United Kingdom and the Republic of Ireland, 1989 1991, 98,127-134.
13. Billings JA, Stoeckle JD: Pelvic examination instruction and the doctor-patient relationship. Journal of Medical Education 1977, 52:8348-49.
14. Boendermaker PM, Faber V, Weijmar Schultz CM: Dealing with difficult sexual questions during consultations: a new training program. Journal of Psychosomatic Obstetrics & Gynecology 2008, 29(2),79-82.
15. Bokken L, Linssen T, Scherpbier A, van der Vleuten C, Rethans J: Feedback by simulated patients in undergraduate medical education: a systematic review of the literature. Medical Education 2009, 43,202-210.
16. Bokken L, Rethans J, van Heurn L, Duvivier R, Scherpbier A, van der Vleuten A: Students' views on the use of real patients and simulated patients in undergraduate medical education. Academic Medicine 2009, 84,958-963.
17. Campbell HS, McBean M, Mandin H, Bryant H: Teaching medical students how to perform a clinical breast examination. Academic Medicine 1994, 12,993-995.

18. Carr SE, Carmody D: Outcomes of teaching medical students core skills in women's health: The pelvic examination educational program. American Journal of Obstetrics and Gynecology 2004, 190,1382-1387.
19. Clements MB, Schmidt KM, Canfield SE, Gilbert SM, Khandewal SR, Koontz BF, Lallas CD, Liauw S, Nguyen PL, Schowalter TN, Trabulsi EJ, Cathro HP, Schenkman NS, Krupski TL: Creation of a novel digital rectal examination evaluation instrument to teach and assess prostate examination proficiency. Journal of Surgical Education 2017, 75(2),434-441.
20. Coleman EA, Hardin SM, Lord JE, Heard JK, Cantrell MJ, Coon SK: General characteristics and experiences of specialized standardized patients: breast teaching associate professionals. Journal of Cancer Education 2002, 17(3),121-123.
21. Coleman EA, Stewart CB, Wilson S, Cantrell MJ, O'Sullivan P, CarthronDO, Wood LC: An evaluation of standardized patients in improving clinical breast examination for military women. Cancer Nursing 2004, 27(6),474-482.
22. Coplan B, Essary AC, Lohenry K, Stoehr JD: An update on the utilization of standardized patients in physician assistant education. J Physician Assist Educ 2008, 19(4),14-19.
23. Costanza ME, Luckman R, Quirk ME, Clemow L, White MJ, Stoddard AM: The effectiveness of using standardized patients to improve community physician skills in mammography counseling and clinical breast exam. Preventive Medicine 1999, 29,241-249.
24. Dabson AM, Magin PJ, Heading G, Pond D: Medical students' experiences learning intimate physical examination skills: A qualitative study. MBC Medical Education 2014, 14(39).
25. Duffy JMN, Chequer S, Braddy A, MylanS, Royuela A, Zamora J, Hayden S, Showell M, Kinnersley P, Chenoy R, Westwood OM, Khan KS, Cushing A: Educational effectiveness of gynaecological teaching associates: a multi-centre randomised controlled trial. BJOG: An International Journal of Obstetrics & Gynaecology 2016, 123(6).
26. Dugoff L, Everett MR, Vontver L, Barley GE: Evaluation of pelvic and breast examination skills of interns in obstetrics and gynecology and internal medicine. Am J Obstet Gynecol 2003, 189(3),655-658.
27. Fairbank C: Men's health: it is imperative to teach scrotal and rectal examination. The Clinical Teacher 2011, 8,101-104.
28. Fairbank C, Reid K, Minsenmay K: Women's experiences of working as a Clinical Teaching Associate teaching sensitive examinations: a qualitative study. Medical Teacher 2015, 37(1),47-52.
29. Fang WL, Hillard PJA, Lindsay RW, Underwood PB: Evaluation of students' clinical and communication skills in performing a gynecologic examination. Journal of Medical Education 1984, 59:758-760.
30. Gerber W, Matthes S, Albanese M: A comparison of instructional techniques: internal-external vs external only. In Annual Conference on Research in Medical Education 1979, 18:59-64.
31. Gilson GJ, George KE, Qualls CM, Sarto GE, Obenshain SS, Boulet J: Assessing clinical competence of medical students in women's health care: Use of the objective structured clinical examination. Obstetrics & Gynecology 1998, 92(6),1038-1043.

32. Godkins TR, Duffy D, Greenwood J, Stanhope, WD: Utilization of simulated patients to teach the 'routine' pelvic examination. Journal of Medical Education 1974, 49:1174-1178.
33. Grankvist O, Olofsson AD, Isaksson R: Can physicians be replaced with gynecological teaching women to train medical students in their first pelvic examination? A pilot study from Northern Sweden. Patient Education and Counseling 2014, 96,50-54.
34. Guenther SM, Laube DW, Matthes S: Effectiveness of the Gynecology Teaching Associate in teaching pelvic examination skills. Journal of Medical Education 1983, 58:67-69.
35. Hale RW, Schiner W: Professional patients: An improved method of teaching breast and pelvic examination. The Journal of Reproductive Medicine 1977, 19(3),163-166.
36. Hendrickx K, De Winter B, Tjalma W, Avonts D, Peeraer G, Wyndaele J: Learning intimate examinations with simulated patients: The evaluation of medical students' performance. Medical Teacher 2009, 31,e139-e147.
37. Hendrickx K, De Winter BY, Wyndaele J Tjalma WAA, Debaene L, Selleslags B, Mast F, Buytaert P, Bossaert L: Intimate examination teaching with volunteers: Implementation and assessment at the University of Antwerp. Patient Education and Counseling 2006,63,47-54.
38. Hendrickx K, De Winter B, Wyndaele J, Tonks A: How medical students are being taught at the University of Antwerp. BMJ 2003, 326(7402)1327.
39. Herbers JE, Wessel L, El-Bayoumi J, Hassan SN, St Onge JE: Pelvic examination training for interns: A randomized controlled trial. Academic Medicine 2003,78,1164-1169.
40. Hillard PJ, Fang WL: Medical students' gynecologic examination skills: Evaluation by Gynecology Teaching Associates. Journal of Reproductive Medicine 1986, 31(6), 491-496.
41. Holzman GB, Singleton D, Holmes TF, Maatsch JL: Initial pelvic examination instruction: the effectiveness of three contemporary approaches. Am J Obstet Gynecol 1977, 129(2):124-129.
42. Howley LD, Dickerson K: Medical students' first male urogenital examination: Investigating the effects of instruction and gender on anxiety. Medical Education Online 2003, 8(1), 436-440.
43. Janjua A, Roberts T, Okeahialam N, Clark TJ: Cost-effective analysis of teaching pelvic examination skills using Gynaecology Teaching Associates (GTAs) compared to manikin models (The CEAT Study). BMJ Open 2018, 8.
44. Janjua A, Smith P, Chu J, Raut, Malick S, Gallos I, Sing R, Irani R, Gupta JK, Parle J, Clark TJ: The effectiveness of gynaecology teaching associates in teaching pelvic examination to medical students: a randomised controlled trial. European Journal of Obstetrics & Gynecology and Reproductive Biology 2017, 210,58-63.
45. Janjua A, Smith P, Clark TJ: A cross-sectional study on teaching pelvic examination in medical schools in the UK (the COTES study). Journal of Obstetrics and Gynaecology 2018, 38(4), 521-515.
46. Jha V, Setna Z, El-Hity A, Quinton ND, Roberts TE: Patient involvement in teaching and assessing intimate examination skills: a systematic review. Medical Education 2010, 44,347-357.

47. Johnson GH, Brown TC, Stenchever MA, Gabert HA, Poulson AM, Warenski JC: Teaching pelvic examination to second-year medical students using programmed patients. Am J Obstet Gynecol 1975, 121(5):714-717.
48. Kamemoto LE, Kane KO, Frattarelli LC: Pelvic examination teaching: linking medical student professionalism and clinical competence. Hawaii Medical Journal 2003, 62,171-172.
49. Kaplan AG, Abdelshehid CS, Alipanah N, Zamanasani T, Lee J, Kolla SB, Sountoulides PG, Graversen J, Lusch A, Kaufmann OG, Louie M, Clayman RV, McDougall EM: Genitourinary exam skills training curriculum for medical students: a follow-up study of comfort and skill utilization. Journal of Endourology 2012, 26(10),1350-1355.
50. Kleinman DE, Hage ML, Hoole AJ, Kowlowitz V: Pelvic examination instruction and experience: A comparison of laywoman-trained and physician-trained students. Academic Medicine 1996, 71(11)1239-1243.
51. Kretzschmar RM: Evolution of the Gynecology Teaching Associate: An educational specialist. Am J Obstet Gynecol 1978, 131:367-372.
52. Kretzschmar RM, Guthrie DS: Why not in every school? Journal of the American Medical Women's Association 1984, 39(2), 43-45.
53. Laube DW, Kretzschmar RM, Guenther SM, Lessner JE, Guthrie D: A clinical skills instruction program: the acute abdomen. Journal of Medical Education 1982; 57:726-728.
54. Legro RS, Gnatuk CL, Kunselman AR, Cain J: Oocyte donors as gynecologic teaching associates. Obstet Gynecol 1999, 93,147-150.
55. Lesserman J, Luke CS: An evaluation of an innovative approach to teaching the pelvic examination to medical students. Women & health 1982, 7(2), 31-42.
56. Livingstone RA, Moodie PF, Ostrow DN: A follow-up study of patient-instructors who teach the pelvic examination. Journal of Medical Education 1980, 55(8): 715-717.
57. Livingstone RA, Ostrow DN: Professional patient-instructors in the teaching of the pelvic examination. Am J Obstet Gynecol 1978, 132:64-67.
58. McBain L, Pullon S, Garrett S, Hoare K: Genital examination training: assessing the effectiveness of an integrated female and male teaching programme. BMC Medical Education 2016, 16,299-306.
59. Muggah HF, Stateson S: The Gynecological Teaching Associates program. The Canadian Nurse 1988, 84(2),28-30.
60. Nelson LH: Use of professional patients in teaching pelvic examinations. Obstetrics and Gynecology 1978, 52(5):630-633.
61. Nensi A, Chande N: A survey of digital rectal examination training in Canadian medical schools. Can J Gastroenterol 2012, 26(7),441-444.
62. Nieman LZ, Kelliher GJ, Sachdeva AK, Cohen D: Evaluation of parallel pelvic/breast and male genital/rectal teaching programs. Journal of the American Medical Women's Association 1994, 49(3)73-77.
63. Nikendei C, Diefenbacher K, Köhl-Hackert N, Lauber H, Huber J, Herrmann-Werner A, Herzog W, Schultz J, Jünger J, Krautter M: Digital rectal examination skills: first training experiences, the motives and attitudes of standardized patients. MBC Medical Education 2015, 15(7).

64. Perlmutter JF, Friedman EA: Use of a live mannequin for teaching physical diagnosis in gynecology. The Journal of Reproductive Medicine 1974, 12(4):163-164.
65. Pickard S, Baraister P, Rymer J, Piper J: Can gynaecology teaching associates provide high quality effective training for medical students in the United Kingdom? Comparative Study. BMJ: 2003,327,1389-1392.
66. Plauché WC, Baugniet-Nebrija W: Students' and physicians' evaluations of Gynecologic Teaching Associate program. Journal of Medical Education 1985, 60:870-875.
67. Popadiuk C, Pottle M, Curran V: itching digital rectal examinations to medical students: An evaluation study of teaching methods. Academic Medicine 2002, 77,1140-1146.
68. Pradhan A, Ebert G, Brug P, Swee D, Ananth CV: Evaluating pelvic examination training: Does faculty involvement make a difference? A randomized controlled trial. Teaching and Learning in Medicine: An International Journal 2010, 22(4), 293-297.
69. Robertson K, Hegarty K, O'Connor V, Gunn J: Women teaching women's health: Issues in the establishment of a clinical teaching associate program for the well woman check. Women & Health 2008,37(4),49-65.
70. Robins LS, Alexander GL, Dicken LL, Belville WD, Zweifler AJ: The effect of a standardized patient instructor experience on students' anxiety and confidence levels performing the male genitorectal examination. Teaching and Learning in Medicine 1997, 9(4), 264-269.
71. Robins LS, Zweifler AJ, Alexander GL, Hengstebeck LL, White CA, McQuillan M, Barclay ML: Using standardized patients for teaching and assessment. Academic Medicine 1997, 72(10)S91-S93.
72. Rochelson BL, Baker DA, Mann WJ, Monheit AG, Stone ML: Use of male and female professional patient teams in teaching physical examination of the genitalia. The Journal of Reproductive Medicine 1985, 30(11), 864-866.
73. Sachdeva AK, Wolfson PJ, Blair PG, Gillum DR, Gracely EJ, Friedman M: Impact of a standardized patient intervention to teach breast and abdominal examination skills to third-year medical students at two institutions. The American Journal of Surgery 1997, 173,320-325.
74. Sarmasoglu S, Dinc L, Elcin M, Tarakcioglu Celik GH, Polonko I: Success of the first gynecological teaching associate program in Turkey. Clinical Simulation in Nursing 2016, 12(6),305-312.
75. Seago BL, Ketchum JM, Willett RM: Pelvic examination skills training with genital teaching associates and a pelvic simulator: Does sequence matter? Sim Healthcare 2012, 7,95-101.
76. Shain RN, Crouch SH, Weinberg PC: Evaluation of the Gynecology Teaching Associate versus pelvic model approach to teaching pelvic examination. Journal of Medical Education 1982, 57, 646-648.
77. Shrestha S, Wijma B, Swahnberg K, Siwe K: Learning pelvic examination with professional patients. J Nepal Med Assoc 2010, 49(177),68-75.
78. Siebeck M, Schwald B, Frey C, Röding S, Stegmann K, Fischer F: Teaching the rectal examination with simulations: effects on knowledge acquisition and inhibition. Medical Education 2011, 45,1025-1031.

79. Silverman RE, Araujo M, Nicholson A: Including gynecological teaching associates' perspectives in women's health exams: lessons for improved communication practices. Health Communication 2012, 27(7),723-725.
80. Siwe K, Berterö C, Wijma B: Unexpected enlightening of a "female world". Male medical students' experiences of learning and performing the first pelvic examination. Sexual & Reproductive Healthcare 2012, 3,123-127.
81. Siwe K, Wijma K: Validation of the Fear of Pelvic Examination Scale (F-PEXS) - measuring students' fear of performing a pelvic examination. Journal of Psychosomatic Obstetrics & Gynecology 2015, 36(1),23-28.
82. Siwe K, Wijma B, Berterö C: 'A stronger and clearer perception of self'. Women's experience of being professional patients in teaching the pelvic examination: a qualitative study. General Gynaecology 2006,113,890-895.
83. Siwe K, Wijma B, Sile'n C, Berterö C: Performing the first pelvic examination: female medical students' transition to examiners. Patient Education and Counseling 2007,69,55-62.
84. Siwe K, Wijma K, Stjernquist M, Wijma B: Medical students learning the pelvic examination: Comparison of outcome in terms of skills between a profes a clinical patient model. Patient Education and Counseling 2007, 68,211-217.
85. Smith PP, Choudhury S, Clark TJ: The effectiveness of gynaecological teaching associates in teaching pelvic examination: a systematic review and meta-analysis. Medical Education 2015, 49,1197-1206.
86. Smith I, Del Bene V, Fleming G, Lancaster C: Long term effects of breast exam teaching unit in physical diagnosis on medical students' practices. Annual Conference on Research in Medical Education 1986, 25:8-13.
87. Sörensdotter R, Siwe K: Touching the private parts: how gender and sexuality norms affect medical students' first pelvic examination. Culture, Health, & Sexuality 2016, 18(11),1295-1308.
88. Steiner E, Austin DF, Prouser NC: Detection and description of small breast masses by residents trained using a standardized clinical breast exam curriculum. J Gen Intern Med 2007, 23(2),129-134.
89. Stenchever MA, Irby D, O'Toole B: A national survey of undergraduate teaching in obstetrics and gynecology. Journal of medical education 1979, 54(6), 486-470.
90. Stillman PL, Regan MB, Philbin M, Haley H: Results of a survey on the use of standardized patients to teach and evaluate clinical skills. Academic Medicine 1990, 65,288-292.
91. Theroux R, Pearce C: Graduate students' experiences with standardized patients as adjuncts for teaching pelvic examinations. American Academy of Nurse Practitioners 2006, 18,429-435.
92. Tolmas HC: Adolescent pelvic examination: An effective practical approach.American Journal of Diseases of Children 1991, 145,1269-1271.
93. Underman K: Playing doctor: simulation in medical school as affective practice. Social Science & Medicine 2015, 136,180-188.
94. Van Ravesteijn H, Hageraats E, Rethans J: Training of the gynaecological examination in the Netherlands. Medical Teacher 2007, 29,e93-e99.

95. Vontver L, Irby D, Rakestraw P, Haddock M, Prince E, Stenchever M: The effects of two methods of pelvic examination instruction on student performance and anxiety. Journal of Medical Education 1980, 55(9): 778-785.
96. Wallis LA, Tardiff K, Deane K: Evaluation of teaching programs for male and female genital examinations. Journal of Medical Education 1983, 58(8):664-666.
97. Wallis LA, Tardiff K, Deane K, Frings J: Teaching associates and the male genitorectal exam. Journal of the American Medical Women's Association 1984, 39(2), 57-58, 62.
98. Wånggren K, Fianu Jonassen A, Andersson S, Pettersson G, Gemzell-Danielsson K: Teaching pelvic examination technique using professional patients: a controlled study evaluating students' skills. Acta Obstetricia et Gynecologica 2010, 89,1293-1303.
99. Wånggren K, Petterson G, Csemiczky G, Gemzell-Danielsson K: Teaching medical students gynaecological examination using professional patients - evaluation of students' skills and feelings. Medical Teacher 2005, 27(2),130-135.
100. Wheeler LA, Burke MM, Ling FW: Nurse-midwife involvement in medical education: the pelvic examination. Journal of nurse-midwifery 1981: 26(1), 34-37.
101. Women' Community Health Center, Inc: Experiences of a pelvic teaching group. Women & Health 1975, 1(4),19-20.
