# Supplementary material for: Implementation and utilization of gynecological teaching associate and male urogenital teaching associate programs: a scoping review
Source: Adv Simul (Lond). 2021 May 20;6:19. doi: 10.1186/s41077-021-00172-2 (PMC8138924; doi:10.1186/s41077-021-00172-2)
Supplement: Supplementary file 2 — Additional file 2. Title of Data: Broad Outcomes of GTA/MUTA Studies. [file 41077_2021_172_MOESM2_ESM.pdf]

Online Supplementary Materials

Table 1. Broad Outcomes of GTA/MUTA Studies

Author(s)	Publication Year	Country	Interventions that GTA/MUTA Programs Were Compared With	GTA or MUTA		Alternative terminology used to describe GTA/MUTA	Number of GTAs/MUTAs	Learner Outcomes				GTA/MUTA Outcomes		Overall Outcomes	
				GTA	MUTA			Competence: External Evaluator	Competence: Self-evaluation	Comfort/confidence	Perception of Program	Program Level	Individual Level	Positive	Negative
Abraham	1995	Australia	Clinic patients, peer exams (learner survey)	x		Gynaecological associate or assistant	-		x		x			x	
Abraham	1998	Australia	-	x		Gynaecological assistant	-				x			x	
Abraham, Chapman, Taylor, McBride, & Boyd	2003	Australia	-	x		Professional patient, Patient	-			x	x			x	
Barnard, Owen, Tyson, & Martin	2011	Australia	-	x		Standard terminology use	-		x	x				x	
Barrett, Zapka, Mazor, & Luckmann	2002	United States	1 vs 2 GTA sessions	x		Standardized patient	-	x		x				x	
Beckmann, Barzansky, Sharf, & Meyers	1988	United States	-	x		Standard terminology use	18					x		x	
Beckmann, Lipscomb, Williford, Bryant, & Ling	1992	United States	Clinic patients, anesthetized patients, task trainers	x		Standard terminology use	-					x		x	
Beckmann & Meyers	1988	United States	-	x		Standard terminology use	18						x	x	
Beckmann, Sharf, Barzansky, & Spellacy	1986	United States	-	x		Standard terminology use	-		x		x			x	
Beckmann, Spellacy, Yonke, Barzansky, & Cunningham	1985	United States and Canada	-	x		Standard terminology use, Live paid pelvic model, Live paid teaching associate	-					x		x	
Behrens, Barnes, Gerber, Albanese, Matthes, & Cangelosi	1979	United States	-		x	Teaching-associate-simulated patient	6		x	x	x			x	
Biggs, Harden, & Howie	1991	United Kingdom, Ireland, Australia, New Zealand	Anesthetized patients, task trainers	x		Standard terminology use	-					x			x
Billings & Stoeckle	1977	United States	-	x		Instructor-model	-					x		x	
Boendermaker, Faber, & Weijmar Schultz	2008	The Netherlands	-	x		Standard terminology use	-					x		x	
Bokken, Linssen, Scherpbier, van der Vleuten, & Rethans	2009	The Netherlands	-	x		Standard terminology use	-					x		x	
Bokken, Rethans, van Heurn, Duvivier, Scherpbier, & van der Vleuten	2009	The Netherlands	Clinic patients	x	x	Standardized patient	-					x		x	
Campbell, McBean, Mandin, & Bryant	1994	Canada	Clinic patients	x		Trained well women teacher	-	x		x				x	
Carr & Carmody	2004	Australia	-	x		Teaching associate	12	x	x	x	x	x		x	

Online Supplementary Materials

Table 1. Broad Outcomes of GTA/MUTA Studies

Author(s)	Publication Year	Country	Interventions that GTA/MUTA Programs Were Compared With	GTA or MUTA		Alternative terminology used to describe GTA/MUTA	Number of GTAs/MUTAs	Learner Outcomes				GTA/MUTA Outcomes		Overall Outcomes	
				GTA	MUTA			Competence: External Evaluator	Competence: Self-evaluation	Comfort/confidence	Perception of Program	Program Level	Individual Level	Positive	Negative
Clements, Schmidt, Canfield, Gilbert, Khandewal, Koontz, Lallas, Liauw, Nguyen, Showalter, Trabulsi, Cathro, Schenkman, & Krupski	2017	United States	-		x	Standard terminology use	12	x						x	
Coleman, Hardin, Lord, Heard, Cantrell & Coon	2002	United States	-	x		Breast teaching associate professional	12						x	x	
Coleman, Stewart, Wilson, Cantrell, O'Sullivan, Carthron, & Wood	2004	United States	-	x		Standardized patient	11		x					x	
Coplan, Essary, Lohenry, & Stoehr	2008	United States	-	x	x	Standardized patient	-					x		x	
Costanza, Luckmann, Quirk, Clemow, White, & Stoddard	1999	United States	-	x		Standardized patient	7	x			x			x	
Dabson, Magin, Heading, & Pond	2014	Australia	-	x		Clinical teaching associate	-			x	x			x	
Duffy, Chequer, Braddy, Mylan, Royuela, Zamora, Hayden, Showell, Kinnersley, Chenoy, Westwood, Khan, & Cushing	2016	England	Task trainer	x		Standard terminology use	-	x		x				x	
Dugoff, Everett, Vontver, & Barley	2003	United States	-	x		Standard terminology use	-	x	x					x	
Fairbank	2011	Australia	-		x	Urological teaching associate	-			x				x	
Fairbank, Reid, & Minzenmay	2015	Australia	-	x		Clinical teaching associate	12						x	x	
Fang, Hillard, Lindsay, & Underwood	1984	United States	Task trainer	x		Standard terminology use	-	x	x	x		x		x	
Gerber, Matthes, & Albanese	1979	United States	Clinic patient		x	Teaching-associate	-	x			x		x	x	
Gilson, George, Qualls, Sarto, Obenshain, & Boulet	1998	United States	-	x		Standardized patient	-	x						x	
Godkins, Duffy, Greenwood, & Stanhope	1974	United States	-	x		Simulated patient	4	x				x		x	
Grankvist, Olofsson, & Isaksson	2014	Sweden	-	x		Gynecological teaching woman	5		x	x	x		x	x	
Guenther, Laube, & Matthes	1983	United States	Clinic patients	x		Standard terminology use	-	x	x	x	x			x	

Online Supplementary Materials

Table 1. Broad Outcomes of GTA/MUTA Studies

Author(s)	Publication Year	Country	Interventions that GTA/MUTA Programs Were Compared With	GTA or MUTA		Alternative terminology used to describe GTA/MUTA	Number of GTAs/MUTAs	Learner Outcomes				GTA/MUTA Outcomes		Overall Outcomes	
				GTA	MUTA			Competence: External Evaluator	Competence: Self-evaluation	Comfort/confidence	Perception of Program	Program Level	Individual Level	Positive	Negative
Hale & Schiner	1977	United States	-	x		Professional patient	8	x	x	x	x			x	
Hendrickx, De Winter, Tjalma, Avonts, Peeraer, & Wyndaele	2009	Belgium	-	x	x	Intimate examination associate	-	x	x					x	
Hendrickx, De Winter, Wyndaele, & Tonks	2003	Belgium	Task trainer	x	x	Intimate examination assistant	20	x	x	x	x		x	x	
Hendrickx, de Winter, Wyndaele, Tjalma, Debaene, Selleslags, Mast, Buytaert, & Bossaert	2006	Belgium	Task trainer	x	x	Intimate examination assistant	20 (10 GTA, 10 MUTA)					x		x	
Herbers, Wessel, El-Bayoumi, Hassan, & St Onge	2003	United States	-	x		Standard terminology use	-	x						x	
Hillard & Fang	1986	United States	-	x		Standard terminology use	-	x	x					x	
Holzman, Singleton, Holmes, & Maatsch	1977	United States	Task trainer	x		Professional simulated patient	-	x	x	x				x	
Howley & Dickerson	2003	United States	-		x	Male teaching associate	-			x				x	
Janjua, Roberts, Okeahialam, & Clark	2018	United Kingdom	Task trainer	x		Standard terminology use	8					x		x	
Janjua, Smith, Chu, Raut, Malick, Gallos, Singh, Irani, Gupta, Parle, & Clark	2017	United Kingdom	Task trainer	x		Standard terminology use	-	x		x	x			x	
Janjua, Smith, & Clark	2018	United Kingdom	Task trainer	x		GTA, Expert patient	-	x	x	x		x		x	
Jha, Setna, Al-Hity, Quinton, & Roberts	2010	United Kingdom	Clinic patients	x	x	Real patient, Standardized patient	-	x	x	x	x	x	x	x	
Johnson , Brown, Stenchever, Gabert, Poulson, & Warenski	1975	United States	-	x		Professional patient	-	x	x		x			x	
Kamemoto, Kane, & Frattarelli	2003	United States	-	x		Professional patient	-					x		x	
Kaplan, Abdelshehid, Alipanah, Zamanasani, Lee, Kolla, Sountoulides, Graversen, Lusch, Kaufmann, Louie, Clayman, & McDougall	2012	United States	-		x	Standardized patient	8	x						x	
Kleinman, Hage, Hoole, & Kowlowitz	1996	United States	Model portraying clinic patient	x		Women's health educational consultants, Standardized patient	-	x						x	
Kretzschmar	1978	United States	-	x		Standard terminology use	6	x			x			x	
Kretzschmar & Guthrie	1984	United States	-	x		Standard terminology use, Teaching associate	-	x	x	x	x	x	x	x	

Online Supplementary Materials

Table 1. Broad Outcomes of GTA/MUTA Studies

Author(s)	Publication Year	Country	Interventions that GTA/MUTA Programs Were Compared With	GTA or MUTA		Alternative terminology used to describe GTA/MUTA	Number of GTAs/MUTAs	Learner Outcomes				GTA/MUTA Outcomes		Overall Outcomes	
				GTA	MUTA			Competence: External Evaluator	Competence: Self-evaluation	Comfort/confidence	Perception of Program	Program Level	Individual Level	Positive	Negative
Laube, Kretzschmar, Guenther, Lessner, & Guthrie	1982	United States	-	x		Standard terminology use	-		x	x	x			x	
Legro, Gnatuk, Kunselman, & Cain	1999	United States	-	x		Standard terminology use	10	x	x	x				x	
Leserman & Luke	1982	United States	-	x		Trained women, Demonstrator, Women's teaching team	-		x	x	x		x	x	
Livingstone, Moodie & Ostrow	1980	Canada	Clinic patients	x		Professional patient-instructor, Simulated patient	-	x				x		x	
Livingstone & Ostrow	1978	Canada	Clinic patients	x		Clinical teaching associate	6	x		x	x	x	x	x	
McBain, Pullon, Garrett & Hoare	2016	New Zealand	-	x	x	Teaching associate	-		x	x	x			x	
Muggah & Stateson	1988	Canada	-	x		Standard terminology use	8	x			x			x	
Nelson	1978	United States	Task trainer	x		Professional patient	2	x		x	x			x	
Nensi & Chande	2012	Canada	Clinic patient, task trainer	x	x	Standardized patient, Urologic teaching assistants, Pelvic teaching assistants	-					x		x	
Nieman, Kelliher, Sachdeva & Cohen	1994	United States	-	x	x	Standard terminology use	5	x	x	x	x	x	x	x	
Nikendei, Diefenbacher, Köhl-Hackert , Lauber, Huber, Herrmann-Werner, Herzog, Schultz, Jünger, & Krautter	2015	Germany	-	x	x	Standard terminology use	4 (2 GTA, 2 MUTA)						x	x	
Perlmutter & Friedman	1974	United States	-	x		Mannequin	1					x		x	
Pickard, Baraitser, Rymer, & Piper	2003	United Kingdom	Clinic patient, task trainer	x		Standard terminology use	6	x				x		x	
Plauché & Baugniet-Nebrija	1985	United States	-	x		Standard terminology use	-					x		x	
Popadiuk, Pottle, & Curran	2002	Canada	Task trainer	x	x	Rectal teaching associate	11			x		x		x	
Pradhan, Ebert, Brug, Swee, & Ananth	2010	United States	Model portraying clinic patient	x		Standard terminology use	6	x		x				x	
Robertson, Hegarty, O'Connor, & Gunn	2008	Australia	-	x		Clinical teaching associate in gynaecology	14					x		x	
Robins, Alexander, Dicken, Belville, & Zweifler	1997	United States	-		x	Standardized patient instructor	-			x	x			x	
Robins, Zweifler, Alexander, Hengstebeck, White, McQuillan, & Barclay	1997	United States	Lecture with demonstration	x		Standardized patient instructor	-	x						x	

Online Supplementary Materials

Table 1. Broad Outcomes of GTA/MUTA Studies

Author(s)	Publication Year	Country	Interventions that GTA/MUTA Programs Were Compared With	GTA or MUTA		Alternative terminology used to describe GTA/MUTA	Number of GTAs/MUTAs	Learner Outcomes				GTA/MUTA Outcomes		Overall Outcomes	
				GTA	MUTA			Competence: External Evaluator	Competence: Self-evaluation	Comfort/confidence	Perception of Program	Program Level	Individual Level	Positive	Negative
Rochelson, Baker, Mann, Monheit, & Stone	1985	United States	-	x	x	Standard terminology use	6 (3 GTA, 3 MUTA)			x	x			x	
Sachdeva, Wolfson, Blair, Gillum, Gracely, & Friedman	1997	United States	-	x		Standard terminology use	-	x						x	
Sarmasoglu, Dinc, Elcin, Tarakcioglu Celik, & Polonko	2016	Turkey	-	x		Standard terminology use	1				x			x	
Seago, Ketchum, & Willett	2012	United States	Task trainer	x		Genital teaching associate	-	x		x				x	
Shain, Crouch, & Weinberg	1982	United States	Task trainer	x		Standard terminology use	-	x		x				x	
Shrestha, Wijma, Swahnberg, & Siwe	2010	Sweden, Nepal	-	x		Professional patient	-					x		x	
Siebeck, Schwald, Frey, Röding, Stegmann, & Fischer	2011	Germany	Task trainer		x	Standardized patient	11 (4 GTA, 7 MUTA)			x				x	
Silverman, Araujo, & Nicholson	2012	United States	-	x		Standard terminology use	-					x		x	
Siwe, Berterö, & Wijma	2012	Sweden	-	x		Professional patient	-			x				x	
Siwe & Wijma	2015	Sweden	-	x		Professional patient	-			x				x	
Siwe, Wijma, & Berterö	2006	Sweden	-	x		Professional patient	13						x	x	
Siwe, Wijma, Sile'n, & Berterö	2007	Sweden	-	x		Professional patient	-			x				x	
Siwe, Wijma, Stjernquist, & Wijma	2007	Sweden	-	x		Professional patient	-		x	x				x	
Smith, Choudhury, & Clark	2015	United Kingdom	-	x		Standard terminology use	-	x		x				x	
Smith, Del Bene, Fleming, & Lancaster	1986	United States	-	x		Teacher	-			x				x	
Sörensdotter & Siwe	2016	Sweden	-	x		GTA, Professional patient	-				x			x	
Steiner, Austin, & Prouser	2007	United States		x		Clinical breast exam surrogate patient, patient instructor	-	x						x	
Stenchever, Irby, & O'Toole	1979	United States	-	x		Professional patient	-					x		x	
Stillman, Regan, Philbin, & Haley	1990	United States	-	x		Standardized patient	-					x		x	
Theroux & Pearce	2006	United States	Peer examination	x		Standardized patient	-			x	x			x	
Tolmas	1991	United States	-	x		Standard terminology use	-				x			x	
Underman	2015	United States	-	x		Standard terminology use	20					x		x	
van Ravesteijn, Hageraats, & Rethans	2007	The Netherlands	-	x		Standard terminology use	-					x		x	

Online Supplementary Materials

Table 1. Broad Outcomes of GTA/MUTA Studies

Author(s)	Publication Year	Country	Interventions that GTA/MUTA Programs Were Compared With	GTA or MUTA		Alternative terminology used to describe GTA/MUTA	Number of GTAs/MUTAs	Learner Outcomes				GTA/MUTA Outcomes		Overall Outcomes	
				GTA	MUTA			Competence: External Evaluator	Competence: Self-evaluation	Comfort/confidence	Perception of Program	Program Level	Individual Level	Positive	Negative
Vontver, Irby, Rakestraw, Haddock, Prince, & Stenchever	1980	United States	Clinic patients	x		Professional patient	6			x				x	
Wallis, Tardiff, & Deane	1983	United States	-	x	x	Women's teaching associate, Male teaching associate	18 (12 GTA, 6 MUTA)				x			x	
Wallis, Tardiff, Deane, & Frings	1984	United States	-	x	x	Male teaching associate, Female teaching associate	6				x			x	
Wånggren, Fianu Jonassen, Andersson, Pettersson, & Gemzell-Danielsson	2010	Sweden	Clinic patients	x		Professional patient, Teaching associate	-	x						x	
Wånggren, Pettersson, Csemiczky, & Gemzell-Danielsson	2005	Sweden	-	x		Professional patient,Teaching associate	14	x	x	x	x			x	
Wheeler, Burke, & Ling	1981	United States	-	x		Patient instructor	19				x	x	x	x	
Women's Community Health Center, Inc	1975	United States	-	x		Pelvic models and instructor	-					x			x
