# Supplementary material for: Implementation and utilization of gynecological teaching associate and male urogenital teaching associate programs: a scoping review
Source: Adv Simul (Lond). 2021 May 20;6:19. doi: 10.1186/s41077-021-00172-2 (PMC8138924; doi:10.1186/s41077-021-00172-2)
Supplement: Supplementary file 3 — Additional file 3. Title of Data: Utilization of GTA/MUTA Programs. [file 41077_2021_172_MOESM3_ESM.pdf]

Online Supplementary Materials

Table 2. Utilization of GTA/MUTA Programs

Author(s)	Publication Year	Learner Type									Number of Learners in Session (if range, lowest number)	Timing within Curriculum							Session Length	Physical Examination Techniques Instructed						Independent or Paired Instructors				Length of Initial Training for GTA/MUTA
		Student				Professional																								
		Medical	Nurse Practitioner	Physician Assistant	Midwife	Physician	Nurse Practitioner	Physician Asssistant	Nurse	Not Addressed		1st year	2nd year	3rd year	4th year	5th year	6th year	Not Addressed		Breast	Pelvic	Rectal (Female)	Prostate/Rectal	Urogenital	Not Addressed	Independent	Paired with Peer	Paired with Faculty	Not Addressed	
Abraham	1995	x									3					x			30 min		x						x			
Abraham	1998	x									-					x	x		-		x					x	x			
Abraham, Chapman, Taylor, McBride, & Boyd	2003	x									-		x						-		x					x				
Barnard, Owen, Tyson, & Martin	2011	x									-			x					-		x								x	
Barrett, Zapka, Mazor, & Luckmann	2002	x									1			x					30 min	x						x				
Beckmann, Barzansky, Sharf, & Meyers	1988	x									6		x	x					3 hours		x						x			24 hours
Beckmann, Lipscomb, Williford, Bryant, & Ling	1992	x									-							x	-						x				x	
Beckmann & Meyers	1988	x									-		x	x					-	x	x						x			
Beckmann, Sharf, Barzansky, & Spellacy	1986	x									3		x	x					1 hr 30 min	x	x						x			
Beckmann, Spellacy, Yonke, Barzansky, & Cunningham	1985	x									-							x	-		x								x	
Behrens, Barnes, Gerber, Albanese, Matthes, & Cangelosi	1979	x									4		x						2 hr 30 min				x	x			x			21 hours
Biggs, Harden, & Howie	1991	x									-							x	-		x								x	
Billings & Stoeckle	1977	x									5		x						2 hours		x						x			
Boendermaker, Faber, & Weijmar Schultz	2008					x					1							x	-		x					x				
Bokken, Linssen, Scherpbier, van der Vleuten, & Rethans	2009									x	-							x	-						x				x	

Online Supplementary Materials

Table 2. Utilization of GTA/MUTA Programs

Author(s)	Publication Year	Learner Type									Number of Learners in Session (if range, lowest number)	Timing within Curriculum							Session Length	Physical Examination Techniques Instructed						Independent or Paired Instructors				Length of Initial Training for GTA/MUTA
		Student				Professional																								
		Medical	Nurse Practitioner	Physician Assistant	Midwife	Physician	Nurse Practitioner	Physician Asssistant	Nurse	Not Addressed		1st year	2nd year	3rd year	4th year	5th year	6th year	Not Addressed		Breast	Pelvic	Rectal (Female)	Prostate/Rectal	Urogenital	Not Addressed	Independent	Paired with Peer	Paired with Faculty	Not Addressed	
Bokken, Rethans, van Heurn, Duvivier, Scherpbier, & van der Vleuten	2009	x									2				x	x			-						x				x	
Campbell, McBean, Mandin, & Bryant	1994	x									-	x	x						60 min	x									x	
Carr & Carmody	2004	x									6					x	x		2 hours		x					x				2 hours
Clements, Schmidt, Canfield, Gilbert, Khandewal, Koontz, Lallas, Liauw, Nguyen, Showalter, Trabulsi, Cathro, Schenkman, & Krupski	2017	x				x					-		x	x	x				-				x			x				
Coleman, Hardin, Lord, Heard, Cantrell & Coon	2002					x					1							x	-	x						x				6 hours
Coleman, Stewart, Wilson, Cantrell, O'Sullivan, Carthron, & Wood	2004					x	x	x	x		1							x	30 min	x						x				
Coplan, Essary, Lohenry, & Stoehr	2008			x							-							x	-		x		x	x					x	
Costanza, Luckmann, Quirk, Clemow, White, & Stoddard	1999					x					3							x	30 min	x						x				6 hours
Dabson, Magin, Heading, & Pond	2014	x									-				x				-	x	x	x	x	x		x				

Online Supplementary Materials

Table 2. Utilization of GTA/MUTA Programs

Author(s)	Publication Year	Learner Type									Number of Learners in Session (if range, lowest number)	Timing within Curriculum							Session Length	Physical Examination Techniques Instructed						Independent or Paired Instructors				Length of Initial Training for GTA/MUTA
		Student				Professional																								
		Medical	Nurse Practitioner	Physician Assistant	Midwife	Physician	Nurse Practitioner	Physician Asssistant	Nurse	Not Addressed		1st year	2nd year	3rd year	4th year	5th year	6th year	Not Addressed		Breast	Pelvic	Rectal (Female)	Prostate/Rectal	Urogenital	Not Addressed	Independent	Paired with Peer	Paired with Faculty	Not Addressed	
Duffy, Chequer, Braddy, Mylan, Royuela, Zamora, Hayden, Showell, Kinnersley, Chenoy, Westwood, Khan, & Cushing	2016	x									4							x	2 hr 30 min		x						x			28 hours
Dugoff, Everett, Vontver, & Barley	2003	x									1			x					30 min	x	x						x			
Fairbank	2011	x									-							x	-				x	x			x			
Fairbank, Reid, & Minzenmay	2015	x	x								3							x	-		x								x	
Fang, Hillard, Lindsay, & Underwood	1984	x									4							x	30 min	x	x						x			
Gerber, Matthes, & Albanese	1979	x									4		x						2 hours				x	x			x			20-25 hours
Gilson, George, Qualls, Sarto, Obenshain, & Boulet	1998	x									1			x					30 min	x	x					x		x		
Godkins, Duffy, Greenwood, & Stanhope	1974			x							-	x							-		x								x	
Grankvist, Olofsson, & Isaksson	2014	x									-					x			1 hr 30 min		x						x			1 day
Guenther, Laube, & Matthes	1983	x									4		x						-	x	x						x			
Hale & Schiner	1977	x									-			x					2 hours	x	x						x			
Hendrickx, De Winter, Tjalma, Avonts, Peeraer, & Wyndaele	2009	x									2					x			60 min	x	x		x	x				x		
Hendrickx, De Winter, Wyndaele, & Tonks	2003	x									2							x	-	x	x		x	x		x				

Online Supplementary Materials

Table 2. Utilization of GTA/MUTA Programs

Author(s)	Publication Year	Learner Type									Number of Learners in Session (if range, lowest number)	Timing within Curriculum							Session Length	Physical Examination Techniques Instructed						Independent or Paired Instructors				Length of Initial Training for GTA/MUTA
		Student				Professional																								
		Medical	Nurse Practitioner	Physician Assistant	Midwife	Physician	Nurse Practitioner	Physician Asssistant	Nurse	Not Addressed		1st year	2nd year	3rd year	4th year	5th year	6th year	Not Addressed		Breast	Pelvic	Rectal (Female)	Prostate/Rectal	Urogenital	Not Addressed	Independent	Paired with Peer	Paired with Faculty	Not Addressed	
Hendrickx, de Winter, Wyndaele, Tjalma, Debaene, Selleslags, Mast, Buytaert, & Bossaert	2006	x									2					x			60 min	x	x		x	x				x		8 hours
Herbers, Wessel, El-Bayoumi, Hassan, & St Onge	2003					x					4							x	-		x						x			
Hillard & Fang	1986	x									4	x		x					30 min	x	x	x					x			
Holzman, Singleton, Holmes, & Maatsch	1977	x									2		x						60 min		x	x					x			
Howley & Dickerson	2003	x									4		x						2 hours				x	x			x			
Janjua, Roberts, Okeahialam, & Clark	2018	x									-							x	2 hours		x						x			24 hours
Janjua, Smith, Chu, Raut, Malick, Gallos, Singh, Irani, Gupta, Parle, & Clark	2017	x									4					x			2 hours		x						x			
Janjua, Smith, & Clark	2018	x									-			x	x	x	x		30 min-3 hours		x								x	
Jha, Setna, Al-Hity, Quinton, & Roberts	2010	x				x					-							x	-	x	x	x	x	x					x	
Johnson , Brown, Stenchever, Gabert, Poulson, & Warenski	1975	x									6		x						-		x					x				2 hours
Kamemoto, Kane, & Frattarelli	2003	x									2		x	x					60 min	x	x						x			
Kaplan, Abdelshehid, Alipanah, Zamanasani, Lee, Kolla, Sountoulides, Graversen, Lusch, Kaufmann, Louie, Clayman, & McDougall	2012	x									1			x					-				x	x				x		

Online Supplementary Materials

Table 2. Utilization of GTA/MUTA Programs

Author(s)	Publication Year	Learner Type									Number of Learners in Session (if range, lowest number)	Timing within Curriculum							Session Length	Physical Examination Techniques Instructed						Independent or Paired Instructors				Length of Initial Training for GTA/MUTA
		Student				Professional																								
		Medical	Nurse Practitioner	Physician Assistant	Midwife	Physician	Nurse Practitioner	Physician Asssistant	Nurse	Not Addressed		1st year	2nd year	3rd year	4th year	5th year	6th year	Not Addressed		Breast	Pelvic	Rectal (Female)	Prostate/Rectal	Urogenital	Not Addressed	Independent	Paired with Peer	Paired with Faculty	Not Addressed	
Kleinman, Hage, Hoole, & Kowlowitz	1996	x									4		x	x					3 hours		x						x			
Kretzschmar	1978	x									2		x	x					3.5 hrs (2nd year), 2.5hrs (3rd year)	x	x						x			6-8 weeks
Kretzschmar & Guthrie	1984	x									-							x	-						x				x	
Laube, Kretzschmar, Guenther, Lessner, & Guthrie	1982	x									4			x					2 hours		x						x			
Legro, Gnatuk, Kunselman, & Cain	1999	x									3			x					-		x							x		
Leserman & Luke	1982	x									4		x						-	x	x						x			
Livingstone, Moodie & Ostrow	1980	x									-		x						-		x						x			
Livingstone & Ostrow	1978	x									2		x						2 hr 30 min	x	x						x			30 hours
McBain, Pullon, Garrett & Hoare	2016	x									4					x			2 hours		x			x				x		1 hour
Muggah & Stateson	1988	x									3			x					-		x						x			
Nelson	1978	x									4		x						-		x							x		
Nensi & Chande	2012	x									-							x	-			x	x						x	
Nieman, Kelliher, Sachdeva & Cohen	1994	x									3			x					-	x	x	x	x	x		x	x			
Nikendei, Diefenbacher, Köhl-Hackert , Lauber, Huber, Herrmann-Werner, Herzog, Schultz, Jünger, & Krautter	2015									x	-							x	-			x	x						x	3 hours
Perlmutter & Friedman	1974	x									4				x			x	-		x					x				

Online Supplementary Materials

Table 2. Utilization of GTA/MUTA Programs

Author(s)	Publication Year	Learner Type									Number of Learners in Session (if range, lowest number)	Timing within Curriculum							Session Length	Physical Examination Techniques Instructed						Independent or Paired Instructors				Length of Initial Training for GTA/MUTA
		Student				Professional																								
		Medical	Nurse Practitioner	Physician Assistant	Midwife	Physician	Nurse Practitioner	Physician Asssistant	Nurse	Not Addressed		1st year	2nd year	3rd year	4th year	5th year	6th year	Not Addressed		Breast	Pelvic	Rectal (Female)	Prostate/Rectal	Urogenital	Not Addressed	Independent	Paired with Peer	Paired with Faculty	Not Addressed	
Pickard, Baraitser, Rymer, & Piper	2003	x									4				x				2 hours		x						x			
Plauché & Baugniet-Nebrija	1985	x									2			x					3 hours	x	x					x				
Popadiuk, Pottle, & Curran	2002	x									2			x					60 min			x	x			x				
Pradhan, Ebert, Brug, Swee, & Ananth	2010	x									2			x					30 min		x					x				16 hours
Robertson, Hegarty, O'Connor, & Gunn	2008	x									4					x			2 hr 30 min	x	x						x			
Robins, Alexander, Dicken, Belville, & Zweifler	1997	x									3		x						45 min			x	x			x				
Robins, Zweifler, Alexander, Hengstebeck, White, McQuillan, & Barclay	1997	x									2	x							30 min	x						x				
Rochelson, Baker, Mann, Monheit, & Stone	1985	x									2	x							-		x		x	x		x				
Sachdeva, Wolfson, Blair, Gillum, Gracely, & Friedman	1997	x									1			x					40 min	x						x				
Sarmasoglu, Dinc, Elcin, Tarakcioglu Celik, & Polonko	2016		x								-							x	45 min	x						x				1 day
Seago, Ketchum, & Willett	2012	x									3		x						1 hr 30 min		x					x				
Shain, Crouch, & Weinberg	1982	x									-		x						-		x	x				x				
Shrestha, Wijma, Swahnberg, & Siwe	2010	x			x	x					3		x		x				2 hr		x						x			

Online Supplementary Materials

Table 2. Utilization of GTA/MUTA Programs

Author(s)	Publication Year	Learner Type									Number of Learners in Session (if range, lowest number)	Timing within Curriculum							Session Length	Physical Examination Techniques Instructed						Independent or Paired Instructors				Length of Initial Training for GTA/MUTA
		Student				Professional																								
		Medical	Nurse Practitioner	Physician Assistant	Midwife	Physician	Nurse Practitioner	Physician Asssistant	Nurse	Not Addressed		1st year	2nd year	3rd year	4th year	5th year	6th year	Not Addressed		Breast	Pelvic	Rectal (Female)	Prostate/Rectal	Urogenital	Not Addressed	Independent	Paired with Peer	Paired with Faculty	Not Addressed	
Siebeck, Schwald, Frey, Röding, Stegmann, & Fischer	2011	x									1			x	x				30 min			x	x			x				
Silverman, Araujo, & Nicholson	2012									x	1							x	-	x	x					x				
Siwe, Berterö, & Wijma	2012	x									6		x						2 hours		x						x			
Siwe & Wijma	2015	x									3		x						2 hours		x								x	
Siwe, Wijma, & Berterö	2006									x	4		x	x					2 hours		x							x		
Siwe, Wijma, Sile'n, & Berterö	2007	x									6		x						2 hours		x							x		
Siwe, Wijma, Stjernquist, & Wijma	2007	x									4			x					2 hours		x							x		
Smith, Choudhury, & Clark	2015									x	-							x	-		x								x	
Smith, Del Bene, Fleming, & Lancaster	1986	x									4		x						1 hr 30 min	x							x			
Sörensdotter & Siwe	2016	x									6			x					-		x							x		
Steiner, Austin, & Prouser	2007	x				x					-	x	x						45 min	x						x				
Stenchever, Irby, & O'Toole	1979	x									-							x	-		x								x	
Stillman, Regan, Philbin, & Haley	1990	x									-		x						-	x	x			x					x	8 hours GTA, 6 hours MUTA
Theroux & Pearce	2006		x								-							x	-		x								x	
Tolmas	1991					x					4							x	-		x					x				
Underman	2015	x									-							x	-						x				x	
van Ravesteijn, Hageraats, & Rethans	2007	x									3							x	3 hours	x	x	x					x			10-36 hours (median 26)

Online Supplementary Materials

Table 2. Utilization of GTA/MUTA Programs

Author(s)	Publication Year	Learner Type									Number of Learners in Session (if range, lowest number)	Timing within Curriculum							Session Length	Physical Examination Techniques Instructed						Independent or Paired Instructors				Length of Initial Training for GTA/MUTA
		Student				Professional																								
		Medical	Nurse Practitioner	Physician Assistant	Midwife	Physician	Nurse Practitioner	Physician Asssistant	Nurse	Not Addressed		1st year	2nd year	3rd year	4th year	5th year	6th year	Not Addressed		Breast	Pelvic	Rectal (Female)	Prostate/Rectal	Urogenital	Not Addressed	Independent	Paired with Peer	Paired with Faculty	Not Addressed	
Vontver, Irby, Rakestraw, Haddock, Prince, & Stenchever	1980	x									3							x	1 hr 45 min		x	x					x	x		
Wallis, Tardiff, & Deane	1983	x									3							x	2 hours	x	x		x	x		x	x			25 hours GTA, 2 sessions MUTA
Wallis, Tardiff, Deane, & Frings	1984	x									8+		x						-	x	x		x	x			x			4 sessions
Wånggren, Fianu Jonassen, Andersson, Pettersson, & Gemzell-Danielsson	2010	x									3							x	2 hours		x					x				
Wånggren, Pettersson, Csemiczky, & Gemzell-Danielsson	2005	x									2							x	1 hr 30 min		x					x				
Wheeler, Burke, & Ling	1981	x									3		x						-		x						x			8 hours
Women's Community Health Center, Inc	1975	x									5		x						-		x						x			
