# Supplementary material for: Implementation and utilization of gynecological teaching associate and male urogenital teaching associate programs: a scoping review
Source: Adv Simul (Lond). 2021 May 20;6:19. doi: 10.1186/s41077-021-00172-2 (PMC8138924; doi:10.1186/s41077-021-00172-2)
Supplement: Supplementary file 4 — Additional file 4. Title of Data: Implementation of GTA/MUTA Programs. [file 41077_2021_172_MOESM4_ESM.pdf]

Online Supplementary Materials

Table 3. Implementation of GTA/MUTA Programs

		Safe Work Environment			Case Development			Training SPs			Program Management			Professional Development		
**Author(s)**	**Publication Year**	**Detailed Explanation**	**Mentioned**	**Not Addressed**	**Detailed Explanation**	**Mentioned**	**Not Addressed**	**Detailed Explanation**	**Mentioned**	**Not Addressed**	**Detailed Explanation**	**Mentioned**	**Not Addressed**	**Detailed Explanation**	**Mentioned**	**Not Addressed**
Abraham	1995			x			x			x			x			x
Abraham	1998			x		x			x			x				x
Abraham, Chapman, Taylor, McBride, & Boyd	2003			x			x			x			x			x
Barnard, Owen, Tyson, & Martin	2011			x			x			x			x			x
Barrett, Zapka, Mazor, & Luckmann	2002			x			x			x			x			x
Beckmann, Barzansky, Sharf, & Meyers	1988	x					x	x			x			x		
Beckmann, Lipscomb, Williford, Bryant, & Ling	1992			x			x			x		x				x
Beckmann & Meyers	1988	x					x			x			x			x
Beckmann, Sharf, Barzansky, & Spellacy	1986			x	x					x			x			x
Beckmann, Spellacy, Yonke, Barzansky, & Cunningham	1985			x			x			x		x				x
Behrens, Barnes, Gerber, Albanese, Matthes, & Cangelosi	1979			x	x				x		x					x
Biggs, Harden, & Howie	1991			x			x			x			x			x

Online Supplementary Materials

Table 3. Implementation of GTA/MUTA Programs

		Safe Work Environment			Case Development			Training SPs			Program Management			Professional Development		
**Author(s)**	**Publication Year**	**Detailed Explanation**	**Mentioned**	**Not Addressed**	**Detailed Explanation**	**Mentioned**	**Not Addressed**	**Detailed Explanation**	**Mentioned**	**Not Addressed**	**Detailed Explanation**	**Mentioned**	**Not Addressed**	**Detailed Explanation**	**Mentioned**	**Not Addressed**
Billings & Stoeckle	1977			x		x			x				x			x
Boendermaker, Faber, & Weijmar Schultz	2008		x		x			x					x			x
Bokken, Linssen, Scherpbier, van der Vleuten, & Rethans	2009			x			x	x			x					x
Bokken, Rethans, van Heurn, Duvivier, Scherpbier, & van der Vleuten	2009			x			x			x			x			x
Campbell, McBean, Mandin, & Bryant	1994			x		x			x			x				x
Carr & Carmody	2004			x		x				x	x					x
Clements, Schmidt, Canfield, Gilbert, Khandewal, Koontz, Lallas, Liauw, Nguyen, Showalter, Trabulsi, Cathro, Schenkman, & Krupski	2017			x			x			x			x			x
Coleman, Hardin, Lord, Heard, Cantrell & Coon	2002			x			x			x		x				x

Online Supplementary Materials

Table 3. Implementation of GTA/MUTA Programs

		Safe Work Environment			Case Development			Training SPs			Program Management			Professional Development		
**Author(s)**	**Publication Year**	**Detailed Explanation**	**Mentioned**	**Not Addressed**	**Detailed Explanation**	**Mentioned**	**Not Addressed**	**Detailed Explanation**	**Mentioned**	**Not Addressed**	**Detailed Explanation**	**Mentioned**	**Not Addressed**	**Detailed Explanation**	**Mentioned**	**Not Addressed**
Coleman, Stewart, Wilson, Cantrell, O'Sullivan, Carthron, & Wood	2004			x		x		x				x				x
Coplan, Essary, Lohenry, & Stoehr	2008			x			x			x			x			x
Costanza, Luckmann, Quirk, Clemow, White, & Stoddard	1999			x	x			x				x				x
Dabson, Magin, Heading, & Pond	2014			x			x			x			x			x
Duffy, Chequer, Braddy, Mylan, Royuela, Zamora, Hayden, Showell, Kinnersley, Chenoy, Westwood, Khan, & Cushing	2016			x			x			x		x				x
Dugoff, Everett, Vontver, & Barley	2003			x		x				x			x			x
Fairbank	2011			x		x				x		x				x
Fairbank, Reid, & Minzenmay	2015		x				x		x			x				x
Fang, Hillard, Lindsay, & Underwood	1984			x	x					x			x			x
Gerber, Matthes, & Albanese	1979			x			x		x		x					x

Online Supplementary Materials

Table 3. Implementation of GTA/MUTA Programs

		Safe Work Environment			Case Development			Training SPs			Program Management			Professional Development		
**Author(s)**	**Publication Year**	**Detailed Explanation**	**Mentioned**	**Not Addressed**	**Detailed Explanation**	**Mentioned**	**Not Addressed**	**Detailed Explanation**	**Mentioned**	**Not Addressed**	**Detailed Explanation**	**Mentioned**	**Not Addressed**	**Detailed Explanation**	**Mentioned**	**Not Addressed**
Gilson, George, Qualls, Sarto, Obenshain, & Boulet	1998			x			x			x			x			x
Godkins, Duffy, Greenwood, & Stanhope	1974			x	x			x			x					x
Grankvist, Olofsson, & Isaksson	2014			x	x			x				x				x
Guenther, Laube, & Matthes	1983			x			x			x			x			x
Hale & Schiner	1977		x		x			x			x					x
Hendrickx, De Winter, Tjalma, Avonts, Peeraer, & Wyndaele	2009			x		x			x				x			x
Hendrickx, De Winter, Wyndaele, & Tonks	2003	x					x	x			x					x
Hendrickx, de Winter, Wyndaele, Tjalma, Debaene, Selleslags, Mast, Buytaert, & Bossaert	2006			x		x			x		x					x
Herbers, Wessel, El-Bayoumi, Hassan, & St Onge	2003			x			x			x			x			x
Hillard & Fang	1986			x			x			x	x					x
Holzman, Singleton, Holmes, & Maatsch	1977			x		x			x			x				x

Online Supplementary Materials

Table 3. Implementation of GTA/MUTA Programs

		Safe Work Environment			Case Development			Training SPs			Program Management			Professional Development		
**Author(s)**	**Publication Year**	**Detailed Explanation**	**Mentioned**	**Not Addressed**	**Detailed Explanation**	**Mentioned**	**Not Addressed**	**Detailed Explanation**	**Mentioned**	**Not Addressed**	**Detailed Explanation**	**Mentioned**	**Not Addressed**	**Detailed Explanation**	**Mentioned**	**Not Addressed**
Howley & Dickerson	2003			x			x			x			x			x
Janjua, Roberts, Okeahialam, & Clark	2018			x			x		x			x				x
Janjua, Smith, Chu, Raut, Malick, Gallos, Singh, Irani, Gupta, Parle, & Clark	2017			x	x				x				x			x
Janjua, Smith, & Clark	2018		x			x				x		x				x
Jha, Setna, Al-Hity, Quinton, & Roberts	2010		x		x				x		x				x	
Johnson , Brown, Stenchever, Gabert, Poulson, & Warenski	1975			x		x		x			x					x
Kamemoto, Kane, & Frattarelli	2003			x			x			x		x				x
Kaplan, Abdelshehid, Alipanah, Zamanasani, Lee, Kolla, Sountoulides, Graversen, Lusch, Kaufmann, Louie, Clayman, & McDougall	2012			x			x			x		x				x
Kleinman, Hage, Hoole, & Kowlowitz	1996			x		x			x				x			x
Kretzschmar	1978	x			x			x			x			x		

Online Supplementary Materials

Table 3. Implementation of GTA/MUTA Programs

		Safe Work Environment			Case Development			Training SPs			Program Management			Professional Development		
**Author(s)**	**Publication Year**	**Detailed Explanation**	**Mentioned**	**Not Addressed**	**Detailed Explanation**	**Mentioned**	**Not Addressed**	**Detailed Explanation**	**Mentioned**	**Not Addressed**	**Detailed Explanation**	**Mentioned**	**Not Addressed**	**Detailed Explanation**	**Mentioned**	**Not Addressed**
Kretzschmar & Guthrie	1984		x			x			x			x				x
Laube, Kretzschmar, Guenther, Lessner, & Guthrie	1982			x	x			x				x				x
Legro, Gnatuk, Kunselman, & Cain	1999			x		x		x			x					x
Leserman & Luke	1982			x		x			x			x				x
Livingstone, Moodie & Ostrow	1980			x			x			x			x			x
Livingstone & Ostrow	1978			x	x			x				x				x
McBain, Pullon, Garrett & Hoare	2016			x	x				x			x				x
Muggah & Stateson	1988			x			x	x				x				x
Nelson	1978		x				x		x		x					x
Nensi & Chande	2012			x			x			x			x			x
Nieman, Kelliher, Sachdeva & Cohen	1994			x	x				x			x				x
Nikendei, Diefenbacher, Köhl-Hackert , Lauber, Huber, Herrmann-Werner, Herzog, Schultz, Jünger, & Krautter	2015		x		x			x			x					x
Perlmutter & Friedman	1974			x			x		x				x			x

Online Supplementary Materials

Table 3. Implementation of GTA/MUTA Programs

		Safe Work Environment			Case Development			Training SPs			Program Management			Professional Development		
**Author(s)**	**Publication Year**	**Detailed Explanation**	**Mentioned**	**Not Addressed**	**Detailed Explanation**	**Mentioned**	**Not Addressed**	**Detailed Explanation**	**Mentioned**	**Not Addressed**	**Detailed Explanation**	**Mentioned**	**Not Addressed**	**Detailed Explanation**	**Mentioned**	**Not Addressed**
Pickard, Baraitser, Rymer, & Piper	2003			x			x		x			x				x
Plauché & Baugniet-Nebrija	1985			x			x			x		x				x
Popadiuk, Pottle, & Curran	2002			x		x			x				x			x
Pradhan, Ebert, Brug, Swee, & Ananth	2010			x		x				x	x					x
Robertson, Hegarty, O'Connor, & Gunn	2008	x			x				x		x					x
Robins, Alexander, Dicken, Belville, & Zweifler	1997			x			x			x			x			x
Robins, Zweifler, Alexander, Hengstebeck, White, McQuillan, & Barclay	1997			x			x			x		x				x
Rochelson, Baker, Mann, Monheit, & Stone	1985			x			x		x			x				x
Sachdeva, Wolfson, Blair, Gillum, Gracely, & Friedman	1997			x		x				x	x					x
Sarmasoglu, Dinc, Elcin, Tarakcioglu Celik, & Polonko	2016			x			x		x				x			x
Seago, Ketchum, & Willett	2012			x			x			x	x					x

Online Supplementary Materials

Table 3. Implementation of GTA/MUTA Programs

		Safe Work Environment			Case Development			Training SPs			Program Management			Professional Development		
**Author(s)**	**Publication Year**	**Detailed Explanation**	**Mentioned**	**Not Addressed**	**Detailed Explanation**	**Mentioned**	**Not Addressed**	**Detailed Explanation**	**Mentioned**	**Not Addressed**	**Detailed Explanation**	**Mentioned**	**Not Addressed**	**Detailed Explanation**	**Mentioned**	**Not Addressed**
Shain, Crouch, & Weinberg	1982			x			x			x			x			x
Shrestha, Wijma, Swahnberg, & Siwe	2010			x			x		x			x				x
Siebeck, Schwald, Frey, Röding, Stegmann, & Fischer	2011			x		x		x				x				x
Silverman, Araujo, & Nicholson	2012			x			x			x			x			x
Siwe, Berterö, & Wijma	2012			x			x		x				x			x
Siwe & Wijma	2015			x			x			x			x			x
Siwe, Wijma, & Berterö	2006			x			x			x			x			x
Siwe, Wijma, Sile'n, & Berterö	2007			x			x		x				x			x
Siwe, Wijma, Stjernquist, & Wijma	2007			x	x			x					x			x
Smith, Choudhury, & Clark	2015			x			x			x			x			x
Smith, Del Bene, Fleming, & Lancaster	1986			x			x			x			x			x
Sörensdotter & Siwe	2016			x			x	x					x			x
Steiner, Austin, & Prouser	2007			x			x			x			x			x
Stenchever, Irby, & O'Toole	1979			x			x			x			x			x

Online Supplementary Materials

Table 3. Implementation of GTA/MUTA Programs

		Safe Work Environment			Case Development			Training SPs			Program Management			Professional Development		
**Author(s)**	**Publication Year**	**Detailed Explanation**	**Mentioned**	**Not Addressed**	**Detailed Explanation**	**Mentioned**	**Not Addressed**	**Detailed Explanation**	**Mentioned**	**Not Addressed**	**Detailed Explanation**	**Mentioned**	**Not Addressed**	**Detailed Explanation**	**Mentioned**	**Not Addressed**
Stillman, Regan, Philbin, & Haley	1990			x			x		x			x				x
Theroux & Pearce	2006			x			x			x			x			x
Tolmas	1991			x			x			x			x			x
Underman	2015			x			x			x			x			x
van Ravesteijn, Hageraats, & Rethans	2007			x		x			x			x				x
Vontver, Irby, Rakestraw, Haddock, Prince, & Stenchever	1980			x			x			x			x			x
Wallis, Tardiff, & Deane	1983			x			x		x				x			x
Wallis, Tardiff, Deane, & Frings	1984			x			x		x			x				x
Wånggren, Fianu Jonassen, Andersson, Pettersson, & Gemzell-Danielsson	2010			x		x				x		x				x
Wånggren, Pettersson, Csemiczky, & Gemzell-Danielsson	2005			x			x		x				x			x
Wheeler, Burke, & Ling	1981	x			x			x			x			x		

Online Supplementary Materials

Table 3. Implementation of GTA/MUTA Programs

		Safe Work Environment			Case Development			Training SPs			Program Management			Professional Development		
**Author(s)**	**Publication Year**	**Detailed Explanation**	**Mentioned**	**Not Addressed**	**Detailed Explanation**	**Mentioned**	**Not Addressed**	**Detailed Explanation**	**Mentioned**	**Not Addressed**	**Detailed Explanation**	**Mentioned**	**Not Addressed**	**Detailed Explanation**	**Mentioned**	**Not Addressed**
Women's Community Health Center, Inc	1975		x			x			x		x				x	
